# USING DRIED BLOOD SPOT ANALYSIS IN OLDER ADULTS WITH MILD COGNITIVE IMPAIRMENT: A FEASIBILITY STUDY

**DOI:** 10.1093/geroni/igae098.3194

**Published:** 2024-12-31

**Authors:** Miranda McPhillips, Junxin Li, Nalaka Gooneratne, Nancy Hodgson

**Affiliations:** Villanova University, Villanova, Pennsylvania, United States; Johns Hopkins School of Nursing, Baltimore, Maryland, United States; University of Pennsylvania, Philadelphia, Pennsylvania, United States; University of Pennsylvania, Philadelphia, Pennsylvania, United States

## Abstract

Dried blood spots (DBS) are whole blood collected on filter paper and dried. We sought to determine the feasibility of using DBS for a virtually delivered intervention, 4 week, Multicomponent Behavioral Sleep Intervention (MBSI) for insomnia in older adults with mild cognitive impairment, during Covid-19. We explored the mechanisms by which MBSI affected sleep via inflammatory biomarkers (IL-6, IL-1ß, TNF-α, and C-reactive protein). Twenty-seven participants were enrolled; 3 dropped out. Participants were mailed supplies and instructions for collecting DBS at 3 study time points (baseline, immediately post-intervention, and 3-month follow-up). Participants were instructed to keep the ﬁlter paper lying ﬂat at room temperature for 24 hours to dry before putting in a biohazard bag with a desiccant and mailing back in a pre-stamped envelope. All samples were labeled, transported, and stored in a -70°C freezer until ready to be analyzed. We used published methods to extract the blood spots in conjunction with Meso Scale Discovery (MSD) multiplex immunoassay for analysis. 20/27 (74%) of participants mailed the DBS cards back at baseline; 14 of those 20 (70%) mailed back DBS cards at timepoint 2 and timepoint 3. Only 10 participants completed all 3 timepoints (10/27; 37%). For all (n=48) samples, CRP and IL-1β values were all within normal limits. For IL-6, 22/48 (46%) values were below the lower limit of detection (LLOD) and 12/48 (25%) were too low to calculate. For TNF-α, 26/48 (54%) values were below LLOD. We will discuss barriers and facilitators to this type of collection and analysis.

